# MicroRNA-1253 suppresses cell proliferation and invasion of non-small-cell lung carcinoma by targeting WNT5A

**DOI:** 10.1038/s41419-017-0218-x

**Published:** 2018-02-07

**Authors:** Meiyue Liu, Yue Zhang, Jie Zhang, Haifeng Cai, Chao Zhang, Zhao Yang, Yi Niu, Huan Wang, Xiaomei Wei, Wei Wang, Peng Gao, Hongmin Li, Jinghua Zhang, Guogui Sun

**Affiliations:** 1 Department of Radiation Oncology, North China University of Science and Technology Affiliated People’s Hospital, Tangshan, 063000 China; 2 Department of Nuclear Medicine, The Second Hospital of Tianjin Medical University, Tianjin, 300211 China; 3 Department of Pathology, North China University of Science and Technology Affiliated People’s Hospital, Tangshan, 063000 China; 4 Department of Breast Oncology, North China University of Science and Technology Affiliated People’s Hospital, Tangshan, 063000 China

## Abstract

MicroRNAs (miRNA) are a class of small, noncoding RNA molecules that regulate the expression of target genes. miRNA dysregulation is involved in carcinogenesis and tumor progression. In this study, we identified microRNA-1253 (miR-1253) as being significantly down-regulated in non-small-cell lung carcinoma (NSCLC) tissues and associated with advanced clinical stage, lymph node metastasis, and poor survival. The enhanced expression of miR-1253 significantly inhibited the proliferation, migration, and invasion of NSCLC cells in vitro. Bioinformatics analyses showed that miR-1253 directly targeted WNT5A (long isoform), which was confirmed using the dual-luciferase reporter assay. The inhibitory effects of miR-1253 on the growth and metastasis of NSCLC cells were attenuated and phenocopied by WNT5A (long) overexpression and knockdown, respectively. Consistent with the in vitro results, subcutaneous tumor and metastatic NSCLC mouse models showed that miR-1253 functions as a potent suppressor of NSCLC in vivo. Taken together, our findings indicated that miR-1253 inhibited the proliferation and metastasis of NSCLC cells by targeting WNT5A (long isoform) and provided new evidence of miR-1253 as a potential therapeutic target in NSCLC.

## Introduction

Lung cancer is a major cause of death, primarily due to its high incidence, aggressiveness, and lack of effective treatments^1^. Non-small-cell lung carcinoma (NSCLC) is the most common type of lung cancer, accounting for approximately 87% of all cases, and it is less sensitive to chemotherapy and/or radiation therapy compared with small-cell lung cancer (SCLC)^2, 3^. Prognosis of lung cancer remains poor due to the high rates of metastasis, recurrence, and drug resistance^4^. The poor outcomes and frequent relapses of lung cancer highlight the urgent need for the development of new screening methods and early biomarkers for the accurate and non-invasive detection metastasis and recurrence^5, 6^. Therefore, there is a need for developing and improving the diagnosis, prevention, and treatment of NSCLC.

MicroRNAs (miRNAs) are small (18–25 nucleotides in length), noncoding RNAs that inhibit translation and/or negatively regulate mRNA stability by binding to the 3’-untranslated region (3’-UTR) of target mRNAs^7^. The “seed” region of a miRNA is the most important region for the recognition of its target mRNA^8^. Depending on the target mRNAs, miRNAs can act as both tumor oncogenes and tumor suppressors. Recent evidence suggests that the dysregulation of miRNAs is involved in a number of biological processes such as development, cell differentiation, cell proliferation, and apoptosis^9, 10^. miRNAs have been shown to play essential roles in the initiation and progression of some cancer types such as lung^11, 12^, breast^13^, and colorectal cancers^14^. Dozens of miRNAs (such as miR-143/145, miR-21, and miR-34) have been shown to play essential roles in lung tumorigenesis by regulating critical oncogenes or tumor suppressors^15–17^. Several studies have shown that miRNAs can be used as diagnostic and prognostic biomarkers. For example, miR-195 expression is reduced in tumor tissues and associated with poor survival outcomes^18^. In colorectal cancer, high levels of miR-135b and low levels of miR-590-5p have been associated with clinical stage and survival^14, 19^.

Recent studies have reported that the expression levels of miR-1253 can predict the overall survival of patients with NSCLC^20^, but the precise molecular mechanisms by which miR-1253 influences NSCLC progression remains largely unknown. Here, we focused on miR-1253 and its signaling pathway to improve our understanding of the pathogenesis of NSCLC and identify promising therapeutic targets.

## Results

### miR-1253 is down-regulated in NSCLC and predicts a poor prognosis

**Fig. 1:**
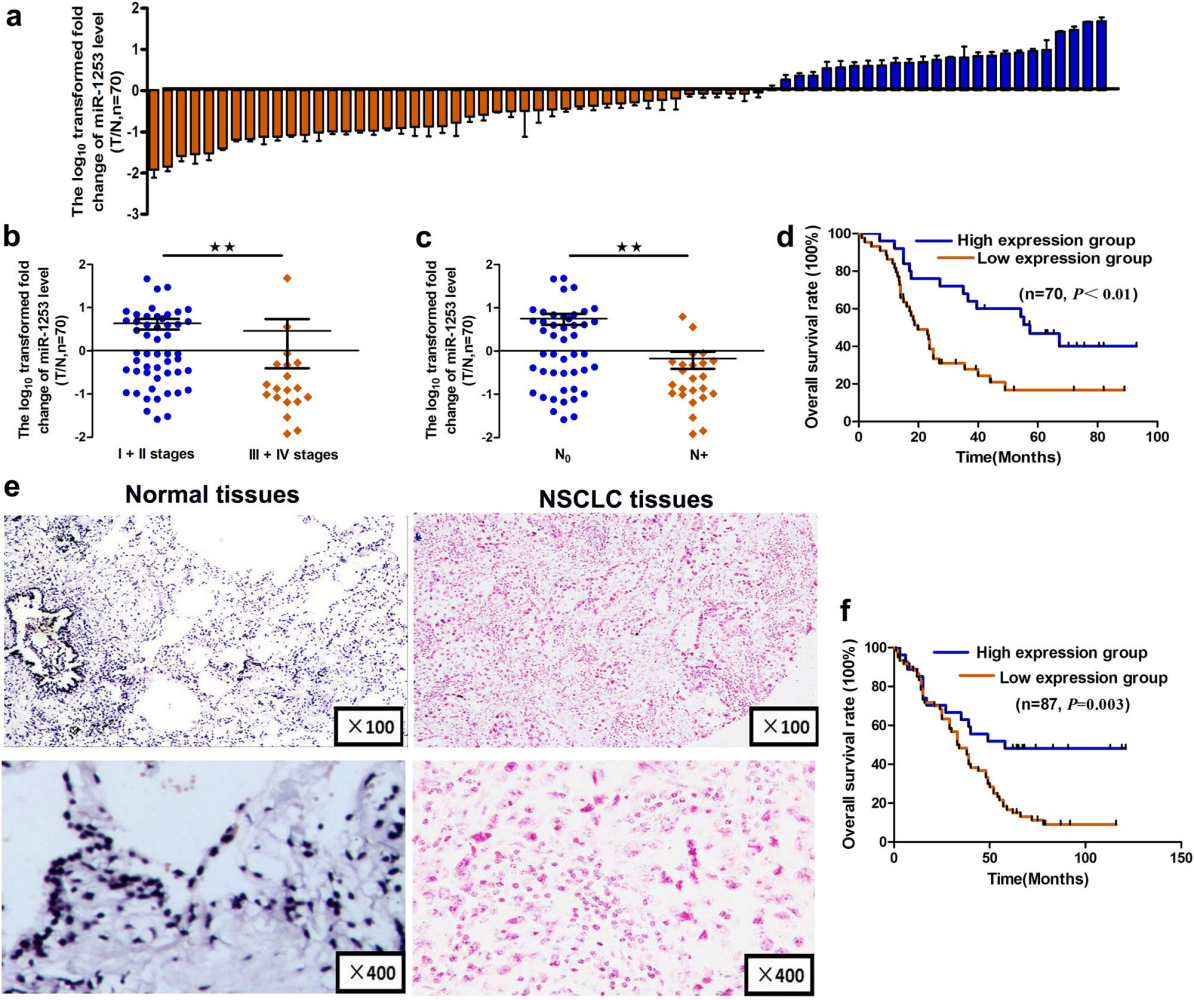
miR-1253 expression in NSCLC and its association with clinical factors. **a** miR-1253 level was measured by qRT-PCR in 70 NSCLC and pair-matched lung tissue samples, and normalized against endogenous U6 RNA. **b**,** c** Expression of miR-1253 in NSCLC was associated with clinical stage (**b**) and lymph node metastasis (**c**). **d** Kaplan–Meier curves depicting overall survival according to the expression of miR-1253 by qRT-PCR. **e** The expression levels of miR-1253 in 87 paired NSCLC and corresponding noncancerous tissues were measured by in situ hybridization. **f** Kaplan–Meier curves depicting overall survival according to high and low miR-1253 expression in 87 patients with NSCLC by in situ hybridization; *p?<?0.01

To explore the expression of miR-1253 in lung cancer, we first analyzed the expression levels of miR-1253 in 70 pairs of human NSCLC tissues and matched non-cancer lung tissues by real-time reverse transcriptase PCR (qRT-PCR). The expression of miR-1253 was normalized to miR-99a and miR-18a-5p. The results also showed that compared to the non-neoplastic lung tissues, miR-1253 expression was significantly down-regulated in the NSCLC tissues (*p?<?*0.05, Fig. 1a, Table 1), especially in the cases with advanced clinical stages (*p?<?*0.05, Fig. 1b, Table 1) and lymph node metastasis (*p?<?*0.05, Fig. 1c, Table 1). On the other hand, the expression of miR-1253 was not associated with age, gender, tumor size, differentiation, and local invasion (all *p?>?*0.05, Table 1). The Kaplan–Meier survival analysis showed that the down-regulation of miR-1253 was associated with poor prognosis in patients with non-small-cell lung cancer (*p*?<?0.05, Fig. 1d).

**Table 1:** Correlation between miR-1253 expression and clinicopathological characteristics in NSCLC patients

Characteristics	Patients with NSCLC (*n?*=?70)			Commercial microarray of NSCLC (*n*?=?87)		
	Low (*n?*=?45)	High (*n*?=?25)	*P* low vs. high	Low (*n*?=?60)	High (*n*?=?27)	*P* low vs. high
Gender						
Male	26 (42.2%)	12 (48.0%)	0.431	26 (43.3%)	13 (48.1%)	0.676
Female	19 (57.8%)	13 (52.0%)		34 (56.7%)	14 (51.9%)	
Age						
<60	23 (51.1%)	12 (48.0%)	0.803	23 (38.3%)	11 (40.7%)	0.831
?60	22 (48.9%)	13 (52.0%)		37 (61.7%)	16 (59.3%)	
Tumor size						
?3?cm	29 (64.4%)	17 (68.0%)	0.764	27 (45.0%)	9 (33.3%)	0.655
>3?cm	16 (35.6%)	8 (32.0%)		37 (55.0%)	18 (66.7%)	
Tumor stage						
T1+T2	32 (71.1%)	22(88.0%)	0.107	44 (73.3%)	22 (81.5%)	0.411
T3+T4	13 (28.9%)	3 (12.0%)		16 (26.7%)	5 (18.5%)	
TNM stage^a^						
I+II	28 (62.2%)	23 (92.0%)	0.007	22 (36.7%)	21 (77.8%)	0.001
III+IV	17 (37.8%)	2 (8.0%)		35 (63.3%)	6 (22.2%)	
Histological grade^a^						
Well/moderate	29 (64.4%)	15 (60.0%)	0.712	42 (70.0%)	17 (63.0%)	0.516
Poor/NS	16 (35.6%)	10 (40.0%)		18 (30.0%)	10 (37.0%)	
Lymph node metastasis^a^						
Negative	23 (51.1%)	23 (92.0%)	0.001	18 (30.0%)	18 (66.7%)	0.003
Positive	22 (48.9%)	2 (8.0%)		30 (70.0%)	6 (33.3%)	

^a^Numbers do not equal to the total number due to missing data

Next, to further validate the clinical significance of miR-1253 in NSCLC patients, in situ hybridization analysis was performed using lung cancer microarray chips. The results showed that the main positive color of miR-1253 was blue or blue purple in the cytoplasm (Fig. 1e). As shown in Table 1, miR-1253 expression was lower in the 87 lung cancer tissues compared with the matching normal lung tissues (*p?<?*0.05). Similar to previous findings, lower miR-1253 levels were detected in patients with advanced clinical stage (compared to early stages) or lymph node metastasis (compared to non-metastatic patients) (Table 1, all *p?<?*0.05). Consistently, patients with higher miR-1253 levels had a lower rate of mortality than patients with lower miR-1253 levels (*p*?<?0.05, Fig. 1f). To normalize the detection method of miR-1253 expression in NSCLC, two other miRNAs, namely, miR-99a and MiR-18a-5p, were assessed, and a similar approach demonstrated that expression miR-99a was significantly down-regulated and miR-18a-5p was obviously up-regulated in tumor tissue samples compared with the controls (Supplementary Figure S1a-b)^21, 22^.

### miR-1253 inhibits the proliferation, colony formation, migration, and invasion of NSCLC cells

**Fig. 2:**
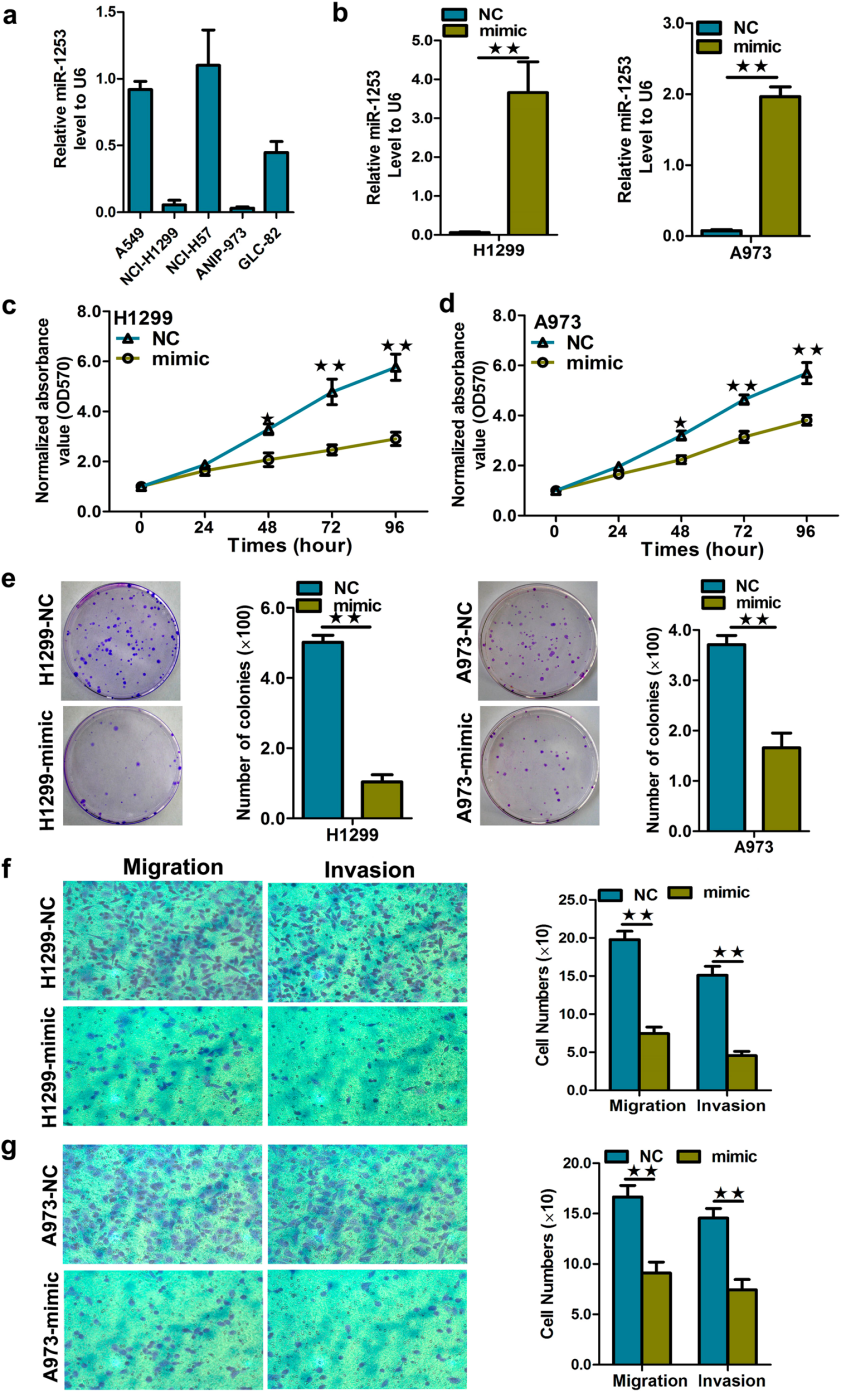
Elevated miR-1253 inhibited cell proliferation, colony formation, and migration. **a** RNA levels of miR-1253 in five NSCLC cell lines. **b** Quantification of miR-1253 levels after miR-1253 mimic transfection into the H1299 and A973 cell lines. **c**,** d** Cell growth was measured by the MTS assay after miR-1253 mimic transfection into the H1299 and A973 cell lines, and the OD_570_ values were normalized to the initial time point (0?h). **e** Representative images and quantification of colony formation after the transfection of miR-1253 mimic into the H1299 and A973 cell lines. **f**,** g** Representative images and quantification of the transwell assays after miR-1253 mimic transfection into H1299 and A973 the cell lines. Data are presented as the mean value?±?SD from triplicate experiments; *p?<?0.05; **p?<?0.01

To assess the role of miR-1253 in NSCLC, we investigated the effects of miR-1253 on proliferation, colony formation, and migration of NSCLC cells. The exogenous expression of miR-1253 was achieved by transfection of miR-1253 mimics into H1299 and A973 cells, which naturally express relatively low levels of miR-1253 (Fig. 2a). The transfection efficiency was verified by qRT-PCR (Fig. 2b). We found that the exogenous expression of miR-1253 inhibited the proliferation, colony formation, migration, and invasion of H1299 and A973 cells (*p*?<?0.05, Fig. 2c–g).

**Fig. 3:**
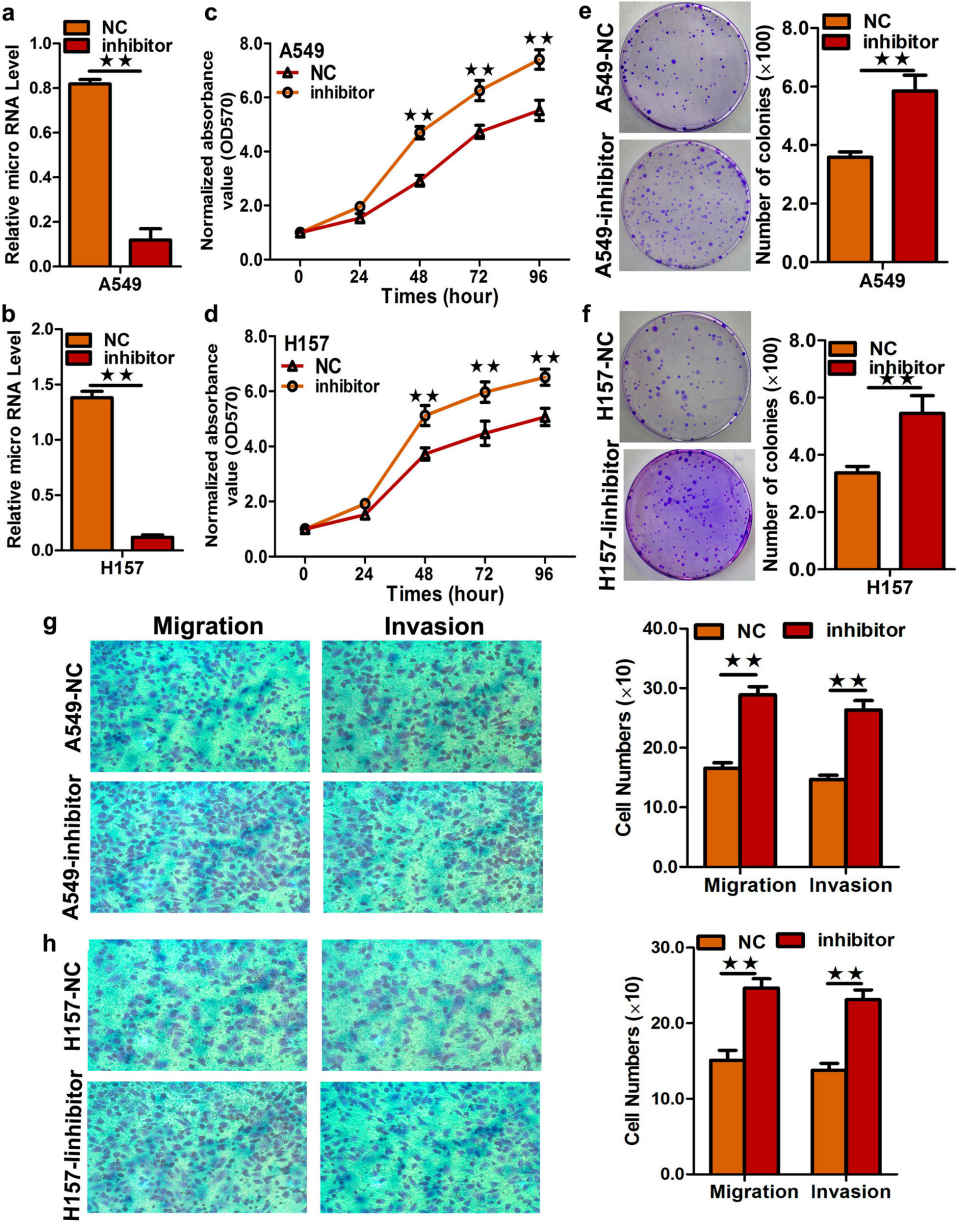
Inhibition of miR-1253 in A549 and H157 cells significantly promoted cell growth, colony formation, and migration. **a**,** b** Quantification of miR-1253 levels after the transfection of miR-1253 inhibitor into the A549 and H157 lines. **c**,** d** Cell growth was measured by MTS assay after the transfection of the miR-1253 inhibitor into the A549 and H157 cell lines, and the OD_570_ values were normalized to the initial time point (0?h). **e**,** f** Representative images and quantification of colony formation after the transfection of the miR-1253 inhibitor into the A549 and H157 cell lines. **g**,** h** Representative images and quantification of the transwell assay after the transfection of the miR-1253 inhibitor into the A549 and H157 cell lines. The experiments were performed in triplicate; *p?<?0.05; **p?<?0.01

We then examined the effect of miR-1253 inhibitors on cell proliferation, migration, and invasion. The A549 and H157 cells were selected because of their high natural miR-1253 expression levels (*p?<?*0.05, Fig. 3a, b). MTS (3-(4, 5-dimethylthiazol-2-yl)-5-(3-carboxymethoxyphenyl)-2-(4-sulfophenyl)-2H-tetazolium) and colony formation assays showed that the growth of A549 and H157 cells was increased by the transfection of miR-1253 inhibitors (*p*?<?0.05, Fig. 3c, d). We further observed that miR-1253 could enhance colony formation in NSCLC cells (*p*?<?0.05, Fig. 3e, f) and that transfection of miR-1253 inhibitors could significantly increase the migration and invasion of A549 and H157 cells (*p*?<?0.05, Fig. 3g, h).

### miR-1253 directly targets the 3’-UTR of WNT5A (long isoform)

**Fig. 4:**
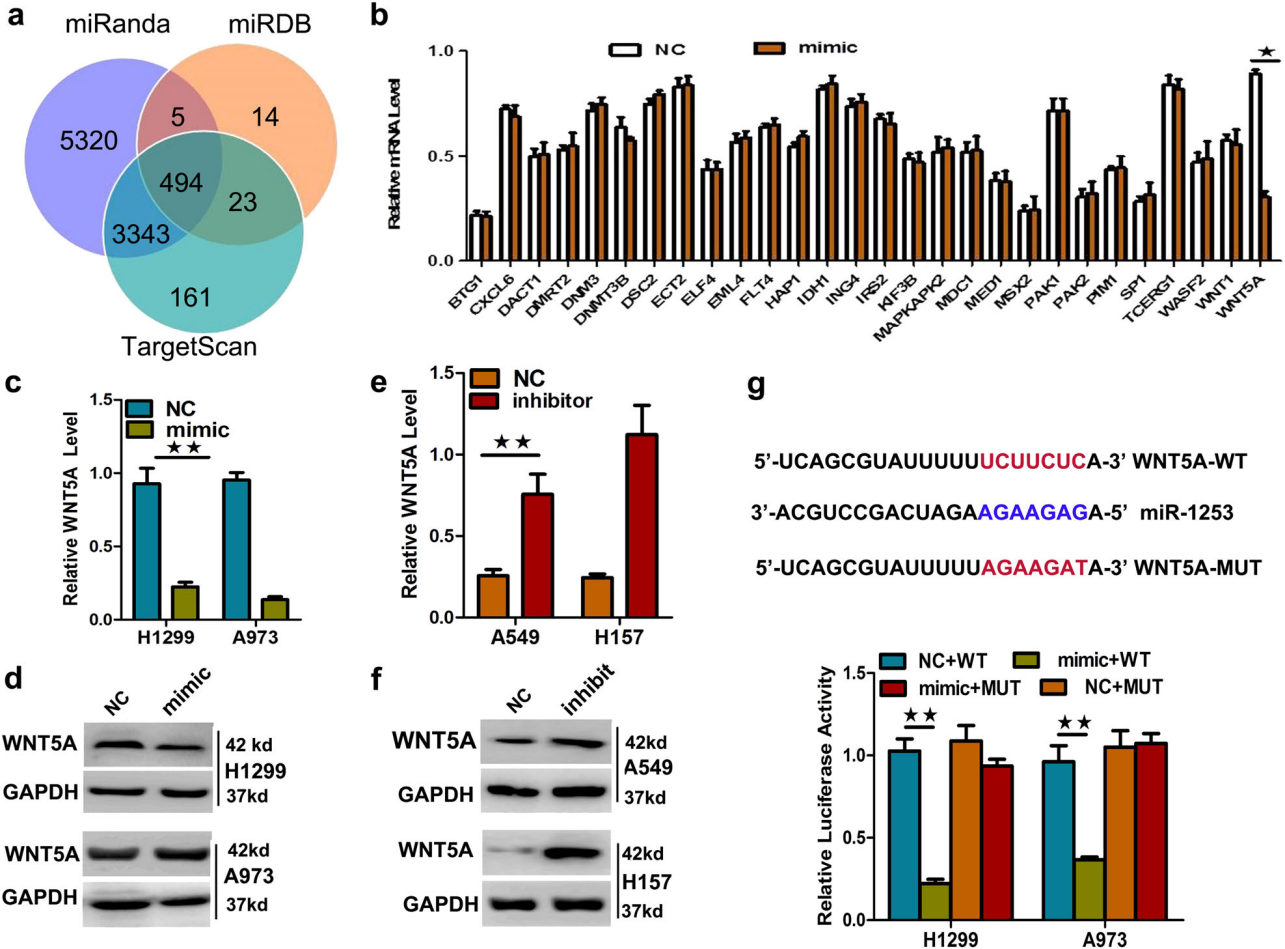
WNT5A was a direct target of miR-1253. **a**,** b** WNT5A was identified as a potential target of miR-1253 after analysis of the down-regulated genes using prediction tools. A total of 494 potential targets were identified by TargetScan (http://www.targetscan.org/), miRDB (http://www.mirdb.org/), and miRanda (http://www.microrna.org/). Among them, the expression pertaining to tumor proliferation, invasion, and metastasis were selected. **c**,** d** Expression levels of the WNT5A mRNA and protein were measured by qRT-PCR and western blot analysis, respectively, using GAPDH as the loading control after the transfection of the miR-1253 mimic into the H1299 and A973 cell lines. **e**, **f** The expression levels of the WNT5A mRNA and protein were measured by qRT-PCR and western blot analysis, respectively, using GAPDH as the loading control, after the transfection of the miR-1253 inhibitors into the A549 and H157 cell lines. **g** Dual-luciferase reporter assay. The relative luciferase activity was normalized to Renilla luciferase activity after the co-transfection of H1299 and A973 cells with the miR-1253 mimic and pmiR-RB-REPORT construct containing the WT or MUT WNT5A 3’-UTR. The experiments were performed in triplicate; *p?<?0.05; *p?<?0.01

To identify the putative mRNA targets of miR-1253, we performed bioinformatics analyses including TargetScan (http://www.targetscan.org/), miRDB (http://www.mirdb.org/), and miRanda (http://www.microrna.org/), and identified 28 candidate targets (Fig. 4a, b, Supplementary Table S1). Among them, the 3’-UTR of WNT5A (long isoform) mRNAs contains sequences that are complementary to miR-1253. We focused on this gene based on its oncogenic role. To verify whether WNT5A (long) was a direct target of miR-1253, we transfected a miR-1253 mimic into cells and observed that this could markedly down-regulate the mRNA and protein levels of WNT5A (long) (*p?<?*0.05, Fig. 4c, d). We also transfected NSCLC cells with inhibitors of miR-1253 to confirm the results of mimic transfection. As expected, down-regulation of miR-1253 using inhibitors could enhance the WNT5A (long) mRNA and protein levels in A549 and H157 cells (*p?<?*0.05, Fig. 4e, f).

Next, we conducted a dual-luciferase reporter assay to confirm the effect of miR-1253 on WNT5A (long isoform). Fragments containing the miR-1253 binding sequence or mutated sequence in the 3’-UTR of the WNT5A (long isoform) mRNA were cloned into the pmiR-RB-REPORT luciferase reporter vector to generate the pmiR-RB-REPORT-WNT5A-3’UTR and pmiR-RB-REPORT-WNT5A-3’UTR-MUT plasmids, respectively. These reporter constructs were co-transfected with miR-1253 mimic or miR-NC into H1299 and A973 cells, and the luciferase activities were measured. It was observed that miR-1253 mimic could significantly suppress the luciferase activity of pmiR-RB-REPORT-WNT5A-3’UTR (*p*?<?0.05, Fig. 4g), while miR-NC had no inhibitory effect on pmiR-RB-REPORT-WNT5A-3’UTR-mut. The inhibition of miR-1253 on pmiR-RB-REPORT-WNT5A-3’UTR is sequence specific because the luciferase activity of pmiR-RB-REPORT-WNT5A-mut was not affected by miR-1253. WNT5A (long isoform) was silenced in 1299 cells and its silencing significantly inhibited cell proliferation, invasion, and migration. Taken together, these results suggested that miR-1253 can directly target the 3’-UTR of WNT5A (long isoform).

**Fig. 5:**
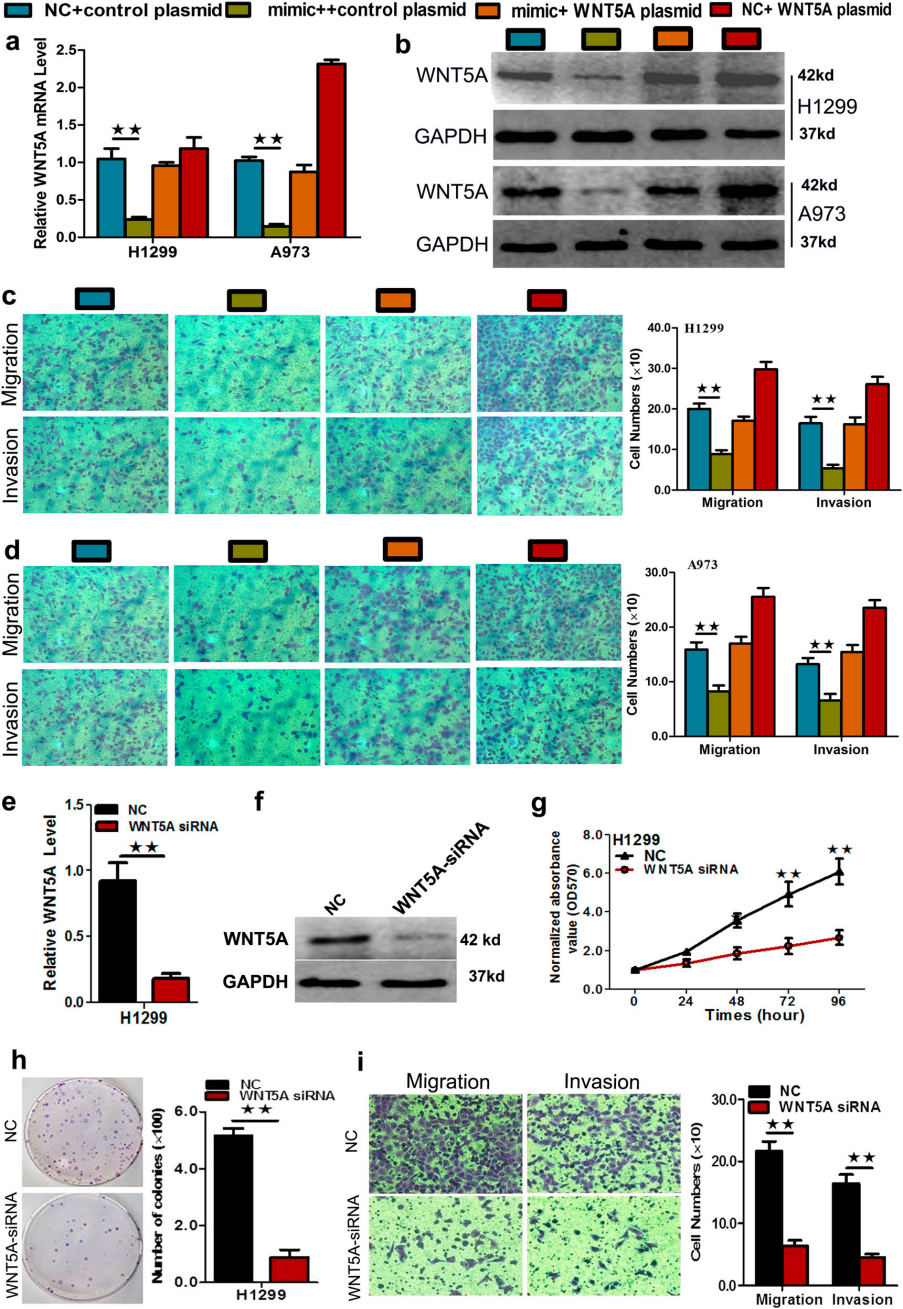
Rescue experiments were performed to confirm that WNT5A is the functional target of miR-1253. **a**,** b** Expression of WNT5A at the mRNA and protein in the H1299 and A973 cell lines transfected with co-transfection of miR-1253 mimic and pEGFP-C1 plasmid containing WNT5A CDS sequence. **c**, **d** Transwell assay in cells co-transfected with the miR-1253 mimic and WNT5A plasmid in the H1299 (**c**) and A973 cell lines (**d**). **e** The expression of WNT5A at the mRNA level post siRNA silencing in 1299 cells. **f** The expression of WNT5A at the protein level post siRNA silencing in 1299 cells. **g**–**i** Representative images and quantification of cell proliferation (**g**), colony formation (**h**), and transwell assay (**i**) after the transfection of WNT5A siRNA into the H1299 cell lines. The experiments were performed in triplicate; .**p?<?0.01

Rescue experiments were performed to confirm that WNT5A (long isoform) is the functional target of miR-1253 in H1299 and A973 cells. Accordingly, WNT5A (long isoform) mRNA and protein levels (endogenous) in the two cell lines were abolished by the transfection of miR-1253 mimic and rescued by the transfection of pEGFP-N1-WNT5A expression constructs (*p*?<?0.05, Fig. 5a, b). The results showed that the migration and invasion of NSCLC cells induced by miR-1253 mimic transfection were reversed by the transfection of pEGFP-N1-WNT5A expression constructs (*p*?<?0.05, Fig. 5c, d). Furthermore, the expression of WNT5A was silenced by small interfering RNA (siRNA) transfection in H1299 cells, which showed that its expression was at the mRNA and protein level was significantly attenuated (*p*?<?0.05, Fig. 5e, f). Post WNT5A silencing, cell proliferation, migration, and invasion ability were significantly inhibited (*p*?<?0.05, Fig. 5g–i). These results further prove that WNT5A may be a downstream target for miR-1253.

### Dysregulation of miR-1253 induced tumorigenesis and metastasis in vivo

**Fig. 6:**
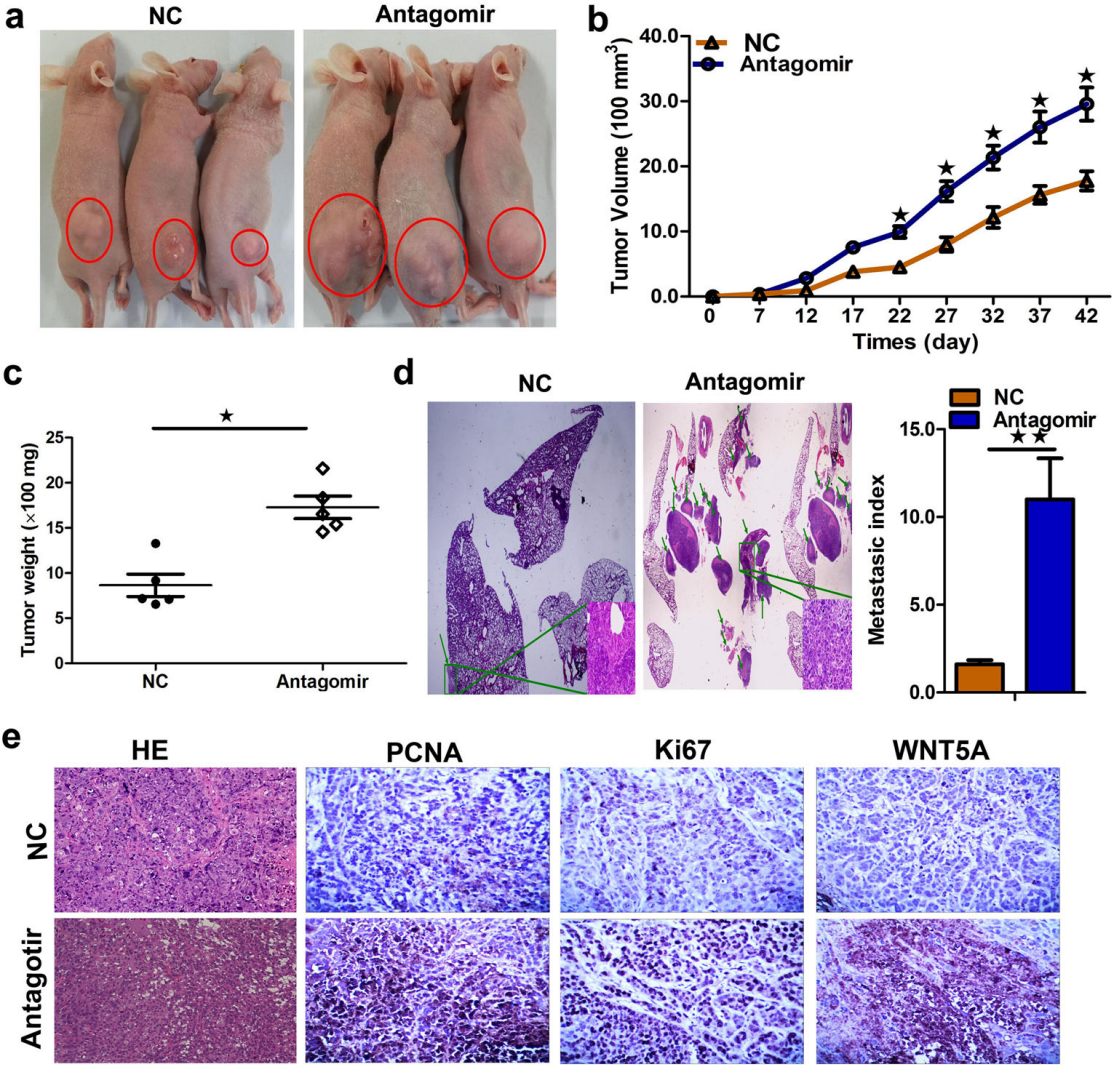
miR-1253 suppressed tumor growth and metastasis in vivo. **a**–**c** A549 cells were subcutaneously injected into nude mice to form solid tumors and treated with either miR-1253 antagomir or miR antagomir NC (*n*?=?5 for each group). **d** The numbers of metastatic nodules were observed and quantified in the lungs of mice treated with miR-1253 antagomir or miR antagomir NC via tail vein injection. **e** Immunohistochemistry of Ki-67, PCNA, and WNT5A in tumor tissues dissected from nude mice treated with miR-1253 antagomir miR or antagomir NC; *p?<?0.05; **p?<?0.01

Previous studies investigating the proliferation-related role of miR-1253 in lung cancer were mainly carried out in vitro. Notwithstanding, the tumor-suppressive effects of miR-1253 can be demonstrated by its ability to inhibit tumorigenesis and/or metastasis in vivo. We established subcutaneous tumor and metastatic tumor models in BALB/c nude mice using A549 cells. After 7 days, miR-1253 antagomir or miR antagomir NC was directly injected into the implanted tumor and the tail vein every 5 days for a total of seven injections. Tumor volume was measured every 5 days until day 42. The results showed that the subcutaneous tumors in the miR-1253 antagomir group grew faster than those in the control group (*p*?<?0.05, Fig. 6a, b). Consistently, the average tumor volume in the miR-1253 down-regulated group was significantly larger than that of the control group (*p*?<?0.05, Fig. 6c). For the metastasis model, 42 days after injection, a higher number of lung metastatic nodules was observed in the miR-1253 down-regulated group compared with that in the control group (*p*?<?0.05, Fig. 6d). Hematoxylin and eosin staining revealed that lung metastases were also markedly increased in the miR-1253 down-regulated group. These data demonstrate a significant association between the dysregulation of miR-1253 and enhanced tumorigenic and metastatic ability of NSCLC cells in vivo. In addition, the proliferative activities of the tumor cells were assessed using immunohistochemistry for proliferating cell nuclear antigen (PCNA) and Ki-67 in formalin-fixed, paraffin-embedded xenograft tumor tissues. PCNA and Ki-67 staining intensities were decreased in the tumors from the miR-1253 antagomir group (Fig. 6e). Moreover, an obvious decrease in WNT5A (long isoform) expression was also observed in the immunostained sections of tumors in the miR-1253 antagomir group compared with those in the control group (Fig. 6e).

## Discussion

During the past few decades, lung cancer has emerged as the leading cause of cancer-related mortality^23^. Although the detection of NSCLC using imaging techniques has rapidly improved, this cannot replace pathological diagnosis, and some metastases may not be identified due to insufficient metabolic/tissue changes^24^. Nevertheless, important metabolic/tissue changes may be responsible for the early detection of NSCLC in some patients, but it is currently impossible to pathologically evaluate every suspicious nodule. A non-invasive and easy sampling strategy that provides reliable information regarding the metastatic potential of NSCLC is urgently required.

miRNAs have been reported to have promising roles in cancer diagnosis and treatment^25–29^. Variations in the expression of microRNAs have been identified in lung cancer tissues compared to their normal counterparts. The reduced expression of microRNAs such as miR-15a and miR-21 has been noted in two common types of NSCLC^30^. In addition, miR-106a, miR-146, miR-155, miR-150, miR-17-3p, miR-191, miR-197, miR-192, miR-21, miR-203, miR-205, miR-210, miR-212, and miR-214 have been reported to be up-regulated in lung cancer^12^. Here, the role of miR-1253 in lung carcinoma and the underlying molecular mechanisms were evaluated.

In this study, we first demonstrated that the expression level of mature miR-1253 was significantly down-regulated in NSCLC tissues and that the levels of miR-1253 were inversely correlated with clinical tumor stage and lymph node metastasis in patients with NSCLC. Despite early diagnosis, almost all patients with NSCLC eventually relapse and experience metastatic disease and death^31^.

Thus, inhibiting metastatic progression to prolong the survival of patients with NSCLC remains a challenge. Our results were consistent with recent findings showing that the expression of tumor-suppressive miRNAs is often down-regulated in NSCLC. For example, miR-195 expression is low in tumor tissues and associated with the clinical stages of NSCLC^18^. In addition, miR-720, which acts as a tumor suppressor by inhibiting cellular migration and invasion, was found to be down-regulated in primary breast carcinoma^32^. A previous study revealed that lymph node metastasis is indicative of advanced disease and poor prognosis; patients with advanced disease usually succumb to the complications caused by metastatic tumors even if they do not suffer from symptoms from the primary tumor^33^. miRNAs have also been extensively investigated as prognostic factors^34^. In addition, our results showed that the lower expression of miR-1253 might be closely associated with worse overall survival. The poor prognosis of patients with lung cancer is usually associated with lymph node metastasis and distant metastasis at diagnosis^35^. Zhou et al.^25^ demonstrated that high serum expression of miR-574-5p was an independent predictor of poor prognosis in patients with SCLC. Similarly, our findings strongly suggest that miR-1253 may play a tumor-suppressive role in NSCLC. Of note, miR-1253 is not conserved, not even in mice.

To better understand the underlying mechanism of miR-1253 in lung cancer, we investigated its biological functions in vitro and in vivo. In the present study, we showed that the ectopic expression of miR-1253 inhibited the proliferation, colony formation, and invasion of NSCLC cells, while the down-regulation of miR-1253 had the opposite effects. Moreover, dysregulation of miR-1253 induced tumor formation and lung metastasis in nude mice. These results indicated that miR-1253 plays a crucial role in the growth and metastasis of NSCLC. Metastasis-related deaths account for approximately 90% of cancer mortality^35^. Accumulating evidence shows that miRNAs participate in tumor growth and/or metastasis, and a growing number of miRNAs have been found to be involved in lung cancer metastasis^36, 37^. Joshi et al.^6^ demonstrated that miR-148a acts as a tumor suppressor and inhibits the migration and invasion of the NSCLC cell line A549. The up-regulation of miR-150 was shown to result in a significant increase in tumor metastasis in vitro and lung metastases in a xenograft model in nude mice^38^. Furthermore, the up-regulation of miR-26b was found to inhibit the migration and invasion of GC cells in vitro and lung metastasis formation in vivo in gastric cancer^39^. These results are consistent with our findings that miR-1253 functions as a tumor suppressor, and thus this miRNA may be used in designing new approaches for cancer therapy by harnessing the mechanisms through which it regulates tumor growth^40^.

A single miRNA can modulate a signaling network by targeting genes with multiple functions. To better understand the underlying tumor-suppressive role of miR-1253 in lung cancer, we aimed to find its potential targets. We found that WNT5A (long isoform) was a direct target of miR-1253. WNT5A (long isoform), an important member of the WNT family, is a critical signaling protein in cells^41^. It has been reported that WNT5A (long isoform) plays an important role in various biological processes, such as cell self-renewal, differentiation, migration, invasion, proliferation, and apoptosis^42^. Accumulating evidence also suggests that WNT5A (long isoform) is involved in diverse pathogenic conditions (including cancer, metabolic diseases, and inflammatory diseases) through multiple WNT signaling receptors^43–45^. In this study, using luciferase reporter assay, qRT-PCR, and western blot analysis, we confirmed that miR-1253 directly targets WNT5A (long isoform). Altered WNT5A (long isoform) expression has been linked to multiple cancers, including breast, gastric, colorectal, and lung cancers^46^. Recently, studies have shown that WNT5A (long isoform) is a target of several miRNAs, including miR-26a^47^, miR-217^48^, miR-374^49^, and miR-590-5p^50^. Zhou et al.^51^ also demonstrated that WNT5A (long isoform) expression is up-regulated in osteosarcoma tissues and cell lines, and that its mRNA expression is negatively correlated with miR-154 expression in osteosarcoma tissues. Nevertheless, a limitation of the luciferase assay in the present study is that the whole 3’-UTR region could not be constructed because it was too long to construct.

In summary, two novel observations were made in the present study. First, to the best of our knowledge, this is the first report showing that miR-1253 is down-regulated in NSCLC and that it is inversely correlated with the expression of WNT5A (long isoform), a novel target in lung cancer. Second, miR-1253 may be an independent prognostic factor in NSCLC. Thus, new diagnostic or therapeutic strategies could be developed by targeting the miR-1253-WNT5A (long isoform) axis.

## Materials and methods

### Human NSCLC tissue samples

Specimens from 70 consecutive patients with NSCLC were retrieved from the pathology department of our hospital. Tissues and matched non-cancer lung tissues were isolated and used for analysis. A tissue microarray representing 87 cases of non-metastatic NSCLC and non-neoplastic lung tissues was purchased from Outdo Biotech (HlugA180Su02, Shanghai, China; http://www.superchip.com.cn/). The clinicopathological characteristics of the patients are summarized in Table 1. None of the patients had received preoperative radiotherapy or chemotherapy. This study was carried out with approval from the hospital ethics committee. Tissue handling was carried out in accordance with institutional policies and approved guidelines for experimental procedures.

### In situ hybridization

miR-1253 LNA probes were purchased from Redlandbio.biomart.cn (Guangzhou, China). In situ hybridization was performed according to the manufacturer’s recommendations under RNase-free conditions.

### Cell lines and cell culture

All cell lines used in this study, including A549, NCI-H1299, NCI-H157, ANIP-973, and GLC-82, were cultured in RPMI medium supplemented with 10% fetal bovine serum at 37?°C in a humidified atmosphere containing 5% CO_2_.

### Isolation of RNA and real-time reverse transcriptase PCR quantification

Total RNA was extracted from frozen tissues or cultured cells using TRIzol total RNA isolation reagent (Invitrogen), according to the manufacturer’s instructions. Complementary DNA was synthesized from total RNA or purified small RNAs using gene-specific primers or random hexamers with the SuperScript III Reverse Transcriptase Kit (Invitrogen), according to the manufacturer’s instructions. PCR was then performed using the Taq polymerase (Takara) with specific primers against WNT5A (long isoform) (forward: 5’-TAAGCCCAGGAGTTGCTTTG-3’ and reverse: 5’-GCAGAGAGGCTGTGCTCCTA-3’) and glyceraldehyde 3-phosphate dehydrogenase (GAPDH) as the internal control (forward: 5’-TCTCTGCTCCTCCTGTTC-3’ and reverse: 5’-GGTTGAGCACAGGGTACTTTATTGA-3’). miRNAs were detected with stem-loop primers purchased from RiboBio (Guangzhou RiboBio Co., Ltd). GAPDH and U6 small nucleolar RNA was used for normalization. qPCR was performed using the QuantiTect SYBR Green PCR Kit (Takara Bio Inc., Otsu, Japan) on a Stepsons Real-Time PCR System (Applied Biosystems, Carlsbad, CA).

### miRNA and siRNA transfection and plasmid construction

The endogenous mature miRNA mimics, inhibitors, and antagomirs were purchased from RiboBio (Guangzhou, China). miRNA mimics, miRNA inhibitors, and miRNA NC were transfected into cells using Lipofectamine 2000 (Invitrogen, Carlsbad, USA), according to the manufacturer’s instructions. After 48?h of transfection, the cells were used for further experiments. The pDonR223-WNT5A plasmid carrying the human WNT5A (long isoform) gene was purchased from Axybio Bio-Tech Co., Ltd (Changsha, China). The complete coding sequence of human WNT5A (long isoform) was amplified from the pDonR223-WNT5A plasmid. PCR products representing the WNT5A (long isoform) coding sequence and pEGFP-N1 plasmid were digested with *Xho*I and* Hin*dIII, and the fragments were purified and ligated with T4 DNA ligase. The ligated product was transformed into Top10 competent cells, and the positive clone was referred to as pEGFP-N1-WNT5A. The oligonucleotides targeting WNT5A and corresponding cholesterol and methylation modified scramble sequences (5’-CATGACATCAGTCGGAGAA-3’) was obtained from RiboBio (RiboBio Co. Ltd., China) and transfected into 1299 cells according to the manufacturer’s protocols.

### Target prediction and luciferase reporter assays

Bioinformatics analysis was performed using specific programs: miRDB (http://www.mirdb.org/), miRanda (http://www.microrna.org), and TargetScan (http://www.targetscan.org/). The 3’-UTR of WNT5A (long isoform) was amplified from human genomic DNA and cloned into the pmiR-RB-REPORT vector (RiboBio, Guangzhou, China) using *Xho*I and *Not*I sites. Similarly, the fragment representing mutant WNT5A (long isoform) 3’-UTR was cloned into the pmiR-RB-REPORT control vector at the same sites. For reporter assays, NSCLC cells were co-transfected with the wild-type reporter plasmid and miR-1253 mimics (miR mimic NC). Firefly and Renilla luciferase activities were measured in cell lysates using the dual-luciferase reporter assay. Luciferase activity was measured 48?h post transfection using the Dual-glo Luciferase Reporter System according to the manufacturer’s instructions (Promega, Madison, WI, USA). Firefly luciferase activity was normalized against Renilla luciferase activity to control for transfection efficiency. WNT5A (long isoform) was also silenced in 1299 cells. The cells were tested for proliferation, invasion, and migration.

### In vitro cell proliferation assays

For the cell proliferation assays, cells were seeded in a 96-well plate (5000 cells/well), and cell proliferation was determined using MTS according to the manufacturer’s instructions. The MTS solution was added (20??l/well) to each well and incubated at 37?°C for 2?h. The optical density of each sample was immediately measured at 570?nm using a microplate reader (Bio-Rad, Hercules, CA, USA).

### Colony formation assay

Cells were transfected with miR-1253 mimic, miR mimic NC, miR-1253 inhibitor, or miR inhibitor NC as described above. After 24?h, the transfected cells were trypsinized, counted, and replated at 1?×?10^3^ cells/10?cm dish. After 10 days, colonies resulting from the surviving cells were fixed with 3.7% methanol, stained with 0.1% crystal violet, and counted. Colonies containing at least 50 cells were scored. Each assay was performed in triplicate.

### Transwell migration/invasion assay

In vitro cell migration assays were performed as previously described using Transwell chambers (8??M pore size; Costar). Cells were allowed to reach about 75–80% confluence and then serum-starved for 24?h. After detachment with trypsin, the cells were washed with phosphate-buffered saline (PBS) and resuspended in serum-free medium. Next, 100??l of cell suspension (5?×?10 ^4^ cells/mL) were added to the upper chamber of the transwell plates. Complete medium was added to the bottom chambers. For the assay, the cells that had not migrated after 24?h were removed from the upper surface of the filters using cotton swabs, but the cells that had migrated were fixed with 5% glutaraldehyde to determine the number of migratory cells. The lower surface of the filters was stained with 0.25% Trypan blue. Images of six different 10× fields were captured from each membrane, and the number of migratory cells was counted. The mean of triplicate assays for each experimental condition was calculated. Similar inserts coated with Matrigel were used to evaluate cell invasive potential using invasion assays.

### Western blot analysis

Cells were washed in PBS and lysed in RIPA lysis buffer supplemented with a protease inhibitor cocktail. Total protein was quantified using a BCA Protein Assay Kit (Beyotime, Nanjing, China), and equal amounts of whole-cell lysates were resolved by SDS-polyacrylamide gel electrophoresis and transferred to polyvinylidene difluoride membranes (Millipore, Germany). The blots were blocked in bovine serum albumin (5% w/v in PBS+0.1% Tween 20) for 1?h at room temperature and immunostained with antibodies at 4?°C overnight. The membranes were probed with anti-WNT5A (long isoform), (ab174963, Cambridge, UK) and anti-GAPDH (ab9485, Abcam) antibodies, followed by incubation with a horseradish peroxidase-conjugated secondary antibodies (goat-anti-mouse IgG (1:2000) and goat-anti-rabbit IgG (1:3000)). Proteins were visualized by Image Reader LAS-4000 (Fujifilm) and analyzed with the Multi Gauge V3.2 software.

### Tumorigenicity and metastasis assay in vivo

All animals were maintained according to the “Guide for the Care and Use of Laboratory Animals” established by the Institute of Laboratory Animal Resources and published by the National Institutes of Health, and according to the Animal Experiment Guidelines of Samsung Biomedical Research Institute. The effect of miR-1253 on the tumorigenic and metastatic potentials of A549 cell were analyzed in subcutaneous tumor and systemic metastasis in vivo models. For the subcutaneous tumor and metastatic tumor model, 3- to 4-week-old BALB/c nude mice were subcutaneously injected in the right hip and intravenously injected through the tail vein with A549 cells (1?×?10^6^ in 100??l of Hanks' balanced salt solution). After 7 days, the subcutaneous tumor and metastatic tumor-bearing nude mice were randomly divided into four groups (*n*?=?5 each). miR-1253 antagomir or miR antagomir NC (NC) was directly injected into the implanted tumor and the tail vein at 1?nmol in 20??L phosphate-buffered saline per mouse every 5 days for a total of seven injections. Tumor size was monitored by measuring the length (L) and width (W) with calipers every 5 days, and the volumes were calculated using: (L?×?W^2^)/2. Mice were killed by cervical dislocation on day 42, and the tumors were excised and snap-frozen for protein and RNA extraction.

### Immunohistochemistry

Tissue slides were incubated for 2?h at 56?°C and deparaffinized. Antigen retrieval was performed by microwaving the slides in citrate buffer for 15?min. After endogenous peroxidase activity was blocked with 3% H_2_O_2_/methanol for 10?min, the sections were incubated with normal goat serum for 20?min to block non-specific antibody binding sites. The sections were incubated with primary antibodies for 1?h at 25?°C, followed by incubation with biotinylated anti-rabbit/mouse IgG and peroxidase-labeled streptavidin for 10?min each. The percentage of cells with cytoplasmic labeling was recorded from two areas of each specimen, and the labeling intensity was estimated as 1+, 2+, or 3+. The immunohistochemistry results were categorized into two groups: the samples without any labeling, 1+ labeling in <25% cells, and 2+ labeling in <5% cells were defined as negative; all the remaining samples were defined as positive.

### Statistical analysis

All data are expressed as the means?±?SD, and all error bars in the figures represent the standard deviation of the mean. Student’s *t*-test, ?^2^ test, and repeated measures analysis of variance were used to determine significance. The log-rank test was used to analyze the effect of clinical variables and miRNA levels on the overall survival of patients. All statistical tests were two sided. *P?<?*0.05 was considered statistically significant. Statistical analyses were performed using SPSS 16.0 (IBM, Armonk, NY, USA).

## Electronic supplementary material

Figure S1

Supplementary Table 1
