# Supplementary figures and images for: MicroRNA-1253 suppresses cell proliferation and invasion of non-small-cell lung carcinoma by targeting WNT5A
Source: Cell Death Dis. 2018 Feb 7;9(2):189. doi: 10.1038/s41419-017-0218-x (PMC5833797; doi:10.1038/s41419-017-0218-x)

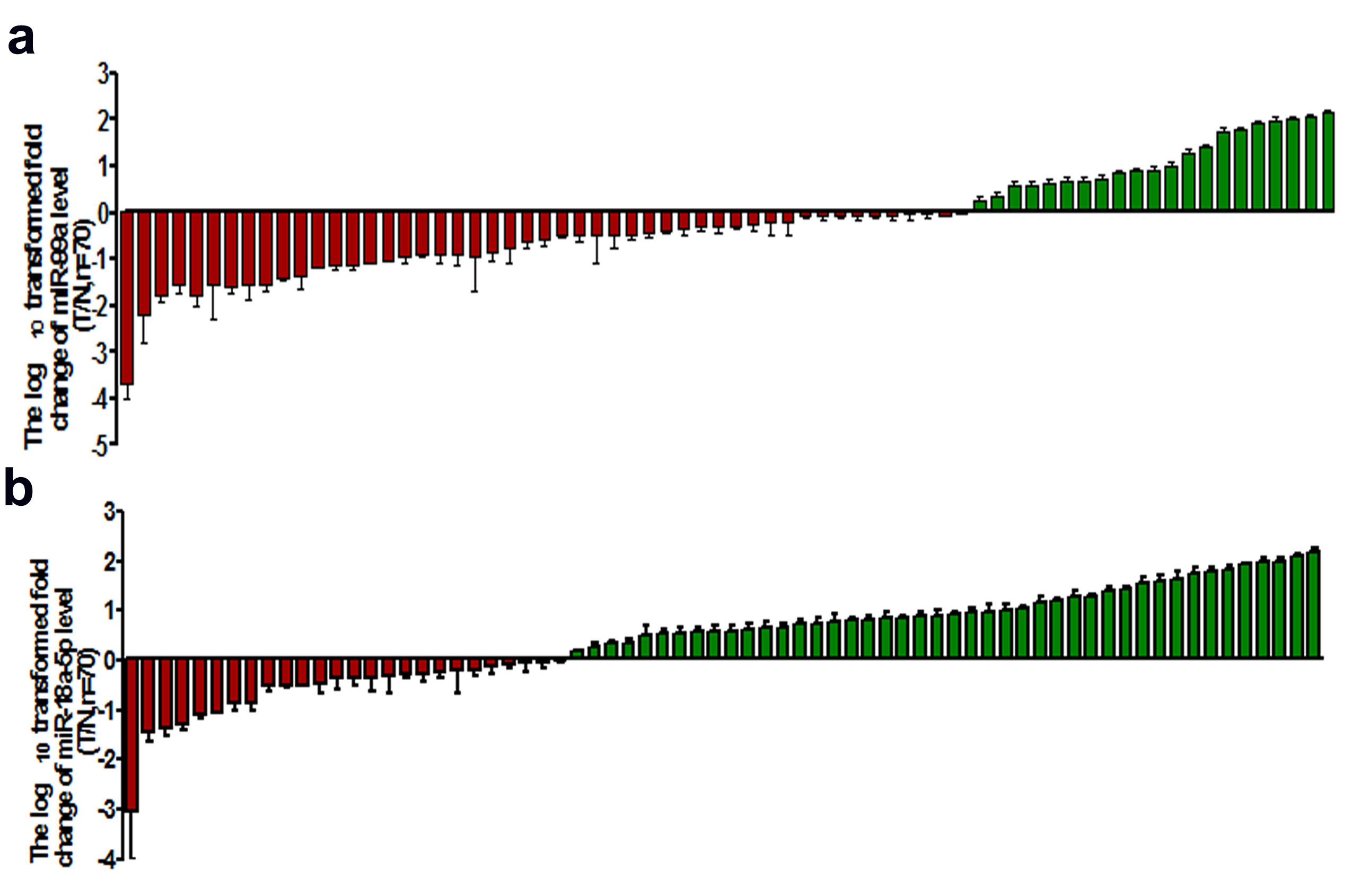

Supplement: Supplementary file 1 — Figure S1 [file 41419_2017_218_MOESM1_ESM.jpg]
