# Supplementary material for: MicroRNA-1253 suppresses cell proliferation and invasion of non-small-cell lung carcinoma by targeting WNT5A
Source: Cell Death Dis. 2018 Feb 7;9(2):189. doi: 10.1038/s41419-017-0218-x (PMC5833797; doi:10.1038/s41419-017-0218-x)
Supplement: Supplementary file 2 — Supplementary Table 1 [file 41419_2017_218_MOESM2_ESM.docx]

**Supplementary Figure Legend**

**Supplementary Figure S1.** The expression of miR-1253 was normalized to miR-99a and miR-18a-5p. (a) The expression levels of miR-1253 in 70 paired NSCLC tissues and corresponding noncancerous tissues were measured by qRT-PCR. (b) Quantitation of miR-3607-3p was performed using qRT-PCR in 70 paired NSCLC tissues and corresponding normal tissues.
